# Screening for Cholesterol-Lowering Probiotics from Lactic Acid Bacteria Isolated from Corn Silage Based on Three Hypothesized Pathways

**DOI:** 10.3390/ijms20092073

**Published:** 2019-04-26

**Authors:** Changlu Ma, Shuwen Zhang, Jing Lu, Cai Zhang, Xiaoyang Pang, Jiaping Lv

**Affiliations:** 1 Institute of Food Science and Technology, Chinese Academy of Agricultural Science, Beijing 100193, China; machanglu@126.com (C.M.); zswmaster@163.com (S.Z.); lujing@caas.cn (J.L.); 2 Department of Food and Bio-engineering, Beijing Vocational College of Agriculture, Beijing 102442, China; 3 Laboratory of Environment and Livestock Products, Henan University of Science and Technology, Luoyang 471023, China; zhangcai@haust.edu.cn; 4 Beijing Advanced Innovation Center for Food Nutrition and Human Health, Beijing Technology & Business University, Beijing 100048, China

**Keywords:** cholesterol removal, NPC1L1 protein, bile salt deconjugation, lactic acid bacteria, probiotic, hypocholesterolemic activity

## Abstract

A total of 85 strains of lactic acid bacteria were isolated from corn silage in this study and analyzed *in vitro* for their cholesterol removal, NPC1L1 protein down-regulation and bile salt deconjugation ability, respectively. Nineteen strains were selected for further analysis for their probiotic potential. Finally, 3 strains showing better probiotic potential were evaluated for their cholesterol-lowering activity in hamsters. The strains showing the greater cholesterol removal and NPC1L1 protein down-regulation activity had no significant effects on serum and hepatic cholesterol levels in hamsters (*p* > 0.05). However, *Lactobacillus plantarum* CAAS 18008 (1 × 10^9^ CFU/d) showing the greater bile salt deconjugation ability significantly reduced serum low-density lipoprotein cholesterol, total cholesterol, and hepatic total cholesterol levels by 28.8%, 21.7%, and 30.9%, respectively (*p* < 0.05). The cholesterol-lowering mechanism was attributed to its bile salt hydrolase activity, which enhanced daily fecal bile acid excretion levels and thereby accelerated new bile acid synthesis from cholesterol in liver. This study demonstrated that the strains showing greater cholesterol removal and NPC1L1 protein down-regulation activity *in vitro* hardly reveal cholesterol-lowering activity *in vivo*, whereas the strains showing greater bile salt deconjugation ability *in vitro* has large potential to decrease serum cholesterol levels *in vivo*.

## 1. Introduction

Cardiovascular disease has become the leading cause of death in many countries worldwide and elevated levels of serum cholesterol have been demonstrated to be the principal cause of this disease [1]. Due to some undesirable side effects from the most commonly used cholesterol-lowering drugs, functional foods and nutraceuticals have recently received more attention to reduce serum cholesterol levels [2]. In the last decade, studies have been focused on cholesterol-lowering effects of probiotics [3], which are live microorganisms that when administered in adequate amounts confer a health benefit on the host [4].

Lactic acid bacteria, especially lactobacilli, are the most widely used probiotic microorganisms [5,6]. Several lactic acid bacterial strains have shown significant cholesterol-cholesterol activity in animals [7,8] and humans [9,10]. However, the exact mechanisms responsible for the cholesterol-lowering activity remain unclear. Three main possible mechanisms have been proposed, which include removing intestinal cholesterol by probiotic cells [11,12], inhibiting small-intestinal cholesterol absorption by the down-regulation of intestinal NPC1L1 protein levels [13], and increasing fecal bile acid excretion levels by bile salt deconjugation that is catalyzed by bile salt hydrolase (BSH) of probiotic cells [10,14]. 

Those probiotic strains of lactic acid bacteria showing cholesterol-lowering activity *in vivo* mainly originated from fermented vegetables [15], fermented dairy products [13], fermented meat products [16], fermented seafood [17], and human or animal feces [18]. However, to our knowledge, no information has been reported on the cholesterol-lowering potential of lactic acid bacterial strains originated from silage. Silage is fermented and high-moisture stored fodder that was widely used to fed to ruminants [19]. During silage fermentation, predominant lactic acid bacteria utilize water-soluble carbohydrates to produce lactic and acetic acids that decreased the pH in silage [20]. The lactic acid bacteria isolated from silage have shown greater antibacterial activity against clinical pathogenic bacteria [21] and gastrointestinal transit tolerance ability [22]. Inoculated silages improved cattle performance, possibly because of probiotic effects of lactic acid bacteria inoculants [23].

The aim of this study was to screen for potential cholesterol-lowering probiotic strains from 85 lactic acid bacterial strains isolated from corn silage based on the three hypothesized pathways *in vitro* and then evaluate their hypocholesterolemic activity in hamsters.

## 2. Results

### 2.1. Cholesterol Removal

**Figure 1:**
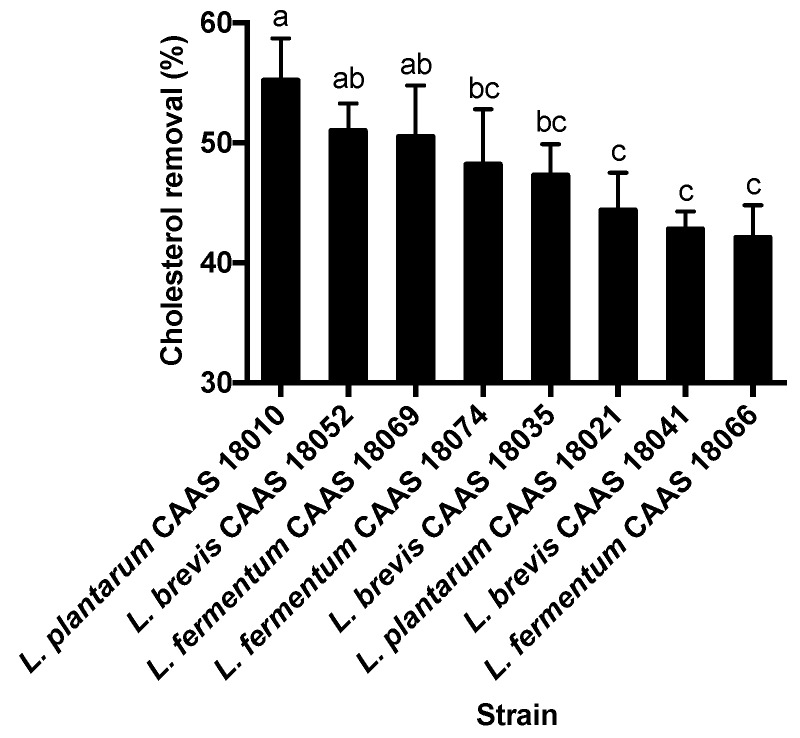
Comparison of lactic acid bacterial strains for cholesterol removal ability. Data are represented as the mean ± SD (*n* = 3). Means not sharing a common letter differ significantly from each other (*p* < 0.05).

All the 85 strains of lactic acid bacteria were able to remove cholesterol from the fermentation broth during the 18-h incubation. Cholesterol removal rates varied among the strains (*p* < 0.05) and ranged from 3.8% to 55.2%. In general, *E. faecium* strains showed lower cholesterol removal rates, and none of the strains were able to remove more than 10% cholesterol from the fermentation broth. The 8 strains showing cholesterol removal rates of more than 40% were selected for further analysis. These strains are listed in Figure 1 in order of decreasing cholesterol removal rates. *L. plantarum* CAAS 18010, *L. brevis* CAAS 18052 and *L. fermentum* CAAS 18069 showed the greatest cholesterol removal ability and removed significantly more cholesterol from the fermentation broth than did *L. plantarum* CAAS 18021, *L. brevis* CAAS 18041 and *L. fermentum* CAAS 18066 (*p* < 0.05).

### 2.2. NPC1L1 Protein Down-Regulation

**Figure 2:**
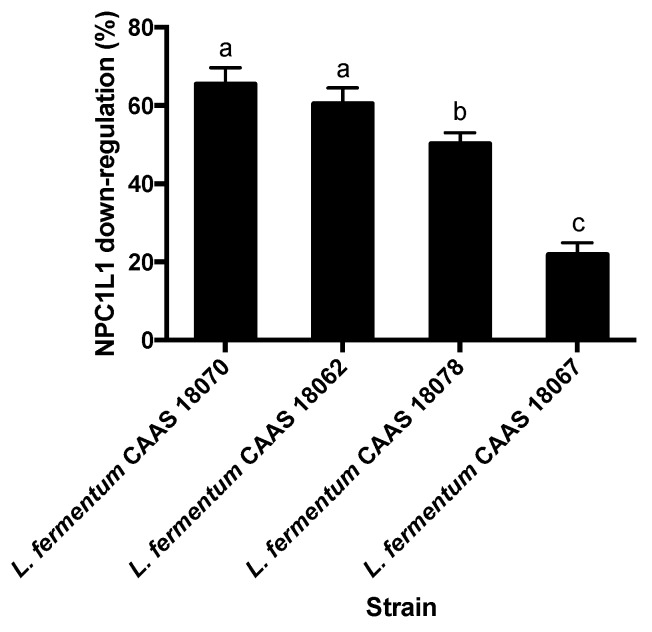
Comparison of lactic acid bacterial strains for NPC1L1 down-regulation ability. Data are represented as the mean ± SD (*n* = 3). Means not sharing a common letter differ significantly from each other (*p* < 0.05).

None of the 85 strains of lactic acid bacteria significantly up-regulated NPC1L1 protein levels of Caco-2 cells (*p* > 0.05). However, 4 strains of *L. fermentum* significantly down-regulated NPC1L1 protein levels of Caco-2 cells (*p* < 0.05) (Figure 2). NPC1L1 protein down-regulation rates varied among the strains (*p* < 0.05) and ranged from 22.5% to 65.5%. *L. fermentum* CAAS 18070 and CAAS 18062 showed the greatest. 

### 2.3. Bile Salt Deconjugation

**Figure 3:**
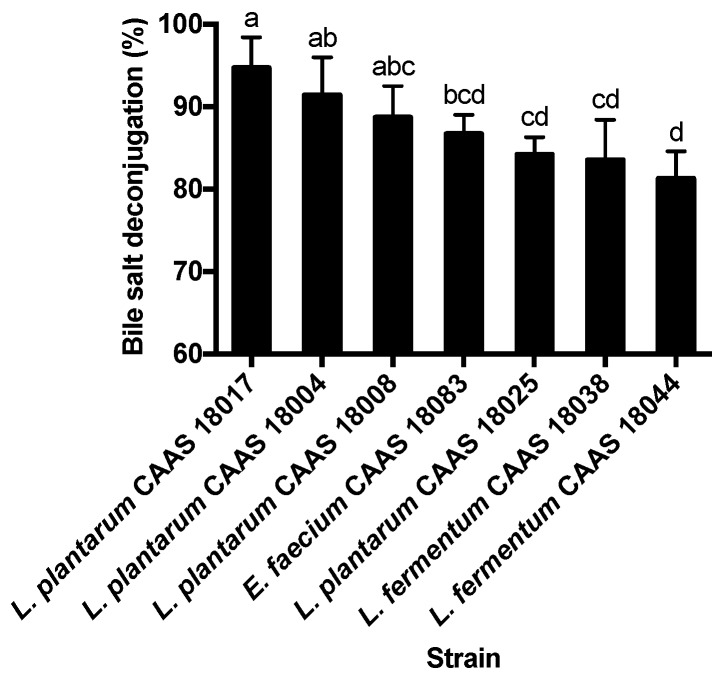
Comparison of lactic acid bacterial strains for bile salt deconjugation ability. Data are represented as the mean ± SD (*n* = 3). Means not sharing a common letter differ significantly from each other (*p* < 0.05).

Fifty-eight strains of the 85 strains of lactic acid bacteria showed bile salt deconjugation ability. These BSH-positive strains consisted of 24 *L. plantarum* strains, 25 *L. brevis* strains, 4 *L. fermentum* strains, and 5 *E. faecium* strains. Bile salt deconjugation ability varied among the 58 BSH-positive strains (*p* < 0.05) and ranged from 8.7 % to 94.7%. The 7 strains showing bile salt deconjugation rates of more than 80% were selected for further analysis. These strains are listed in Figure 3 in order of decreasing bile salt deconjugation rates. *L. plantarum* CAAS 18017, CAAS 18004, and CAAS 18008 showed the greatest bile salt deconjugation ability, while *E. faecium* CAAS 18083, *L. plantarum* CAAS 18025 and *L. brevis* CAAS 18038 and CAAS 18044 showed the least bile salt deconjugation ability. 

### 2.4. Basic Probiotic Properties

The basic probiotic properties, including acid tolerance, bile tolerance, and adhesion ability to Caco-2 cells [24], were further determined for the 8 strains showing the greater cholesterol removal ability, the 4 strains showing NPC1L1 down-regulation activity, and the 7 strains showing the greater bile salt deconjugation ability (Table 1).

Among the 8 strains showing the greater cholesterol removal ability, *L. plantarum* CAAS 18021, *L. fermentum* CAAS 18066, and *L. fermentum* CAAS 18074 were significantly less acid tolerant than the other strains (*p* < 0.05); *L. plantarum* CAAS 18010, *L. fermentum* CAAS 18069 and *L. brevis* CAAS 18035 were significantly less bile tolerant than the other strains (*p* < 0.05). Therefore, these strains were eliminated. The remaining two strains (*L. brevis* CAAS 18041 and CAAS 18052) did not differ significantly in their acid and bile tolerance and adhesion ability (*p* > 0.05), but the latter showed significantly greater cholesterol removal ability than the former (*p* < 0.05) (Figure 1). Therefore, *L. brevis* CAAS 18052 was selected for further animal feeding trail.

Among the 4 strains showing NPC1L1 protein down-regulation activity, *L. fermentum* CAAS 18062 was the least acid tolerant; *L. fermentum* CAAS 18067 was the least adherent to Caco-2 cells; *L. fermentum* CAAS 18070 was the least bile tolerant. Therefore, these strains were eliminated. The remaining 1 strain (*L. fermentum* CAAS 18078) was selected for further animal feeding trail.

Among the 7 strains showing the greater bile salt deconjugation ability, *L. plantarum* CAAS 18004 was the least bile tolerant; *L. plantarum* CAAS 18017, CAAS 18025 and *E. faecium* CAAS 18083 were the least adherent to Caco-2 cells; *L. brevis* CAAS 18038 was the least acid tolerant. Therefore, these strains were eliminated. The remaining 2 strains (*L. plantarum* CAAS 18008 and *L. brevis* CAAS 18044) did not differ significantly in their acid and bile tolerance (*p* > 0.05), but the former showed greater adhesion to Caco-2 cells and bile salt deconjugation than the latter. For this reason, *L. plantarum* CAAS 18008 was selected for further animal feeding trail.

### 2.5. Serum and Hepatic Cholesterol

**Figure 4:**
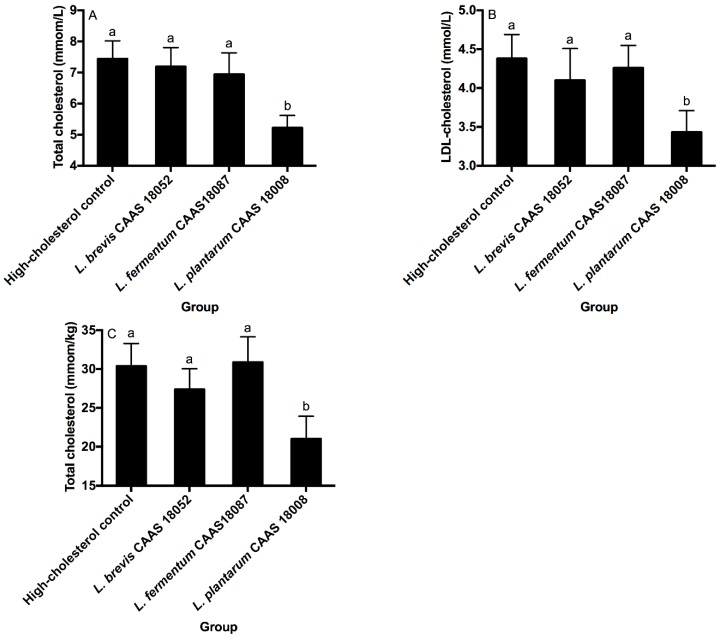
Serum total cholesterol (**A**), LDL-cholesterol (**B**) and hepatic total cholesterol (**C**) levels of the different groups. Data are represented as the mean ± SD (*n* = 8). Means not sharing a common letter differ significantly from each other (*p* < 0.05).

*L. brevis* CAAS 18052, *L. fermentum* CAAS 18078, and *L. plantarum* CAAS 18008 did not significantly affect serum HDL-cholesterol levels in hamsters (*p* > 0.05, data not shown). *L. brevis* CAAS 18052 and *L. fermentum* CAAS 18078 also did not significantly affect serum LDL-cholesterol, total cholesterol, and hepatic total cholesterol levels in hamsters (*p* > 0.05) (Figure 4A–C). However, *L. plantarum* CAAS 18008 significantly decreased serum LDL-cholesterol, total cholesterol and hepatic total cholesterol levels in hamsters by 28.8%, 21.7%, and 30.9%, respectively (*p* < 0.05).

### 2.6. Fecal Sterol Excretion

**Figure 5:**
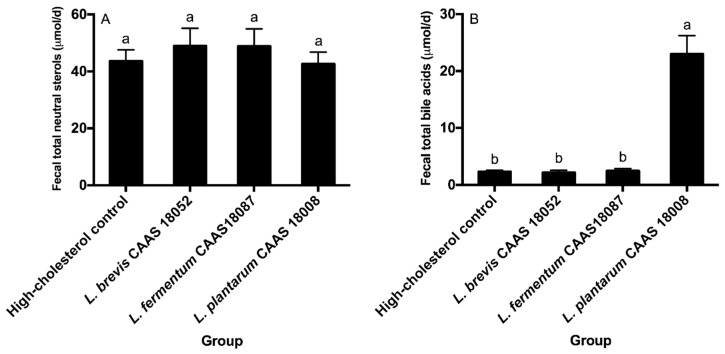
Daily excretion levels of fecal total neutral sterols (**A**) and total bile acids (**B**) of the different groups. Data are represented as the mean ± SD (*n* = 8). Means not sharing a common letter differ significantly (*p* < 0.05).

Daily fecal excretion levels of total neutral sterols and total bile acids of the different groups are shown in Figure 5. *L. brevis* CAAS 18052, *L. fermentum* CAAS 18078, and *L. plantarum* CAAS 18008 did not significantly affect daily fecal total neutral sterol excretion levels in hamsters (*p* > 0.05) (Figure 5A). *L. brevis* CAAS 18052 and *L. fermentum* CAAS 18078 also did not significantly affect daily fecal total bile acid excretion levels in hamsters (*p* > 0.05) (Figure 5B). However, *L. plantarum* CAAS 18008 significantly enhanced daily fecal total bile acid excretion level in hamsters by 9.0 times (*p* < 0.05).

### 2.7. Small Intestinal NPC1L1 Protein and Hepatic Cholesterol-7α-Hydroxylase (CYP7A1)

**Figure 6:**
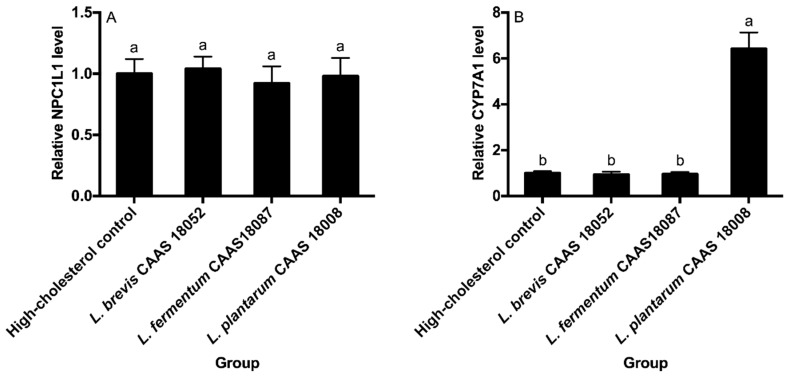
Small intestinal NPC1L1 protein (**A**) and hepatic CYP7A1 (**B**) levels of the different groups. Data are represented as the mean ± SD (*n* = 8). Means not sharing a common letter differ significantly from each other (*p* < 0.05).

The small intestinal NPC1L1 protein and hepatic CYP7A1 levels of the different groups are shown in Figure 6. *L. brevis* CAAS 18052, *L. fermentum* CAAS 18078, and *L. plantarum* CAAS 18008 did not significantly affect the small intestinal NPC1L1 protein levels (*p* > 0.05) (Figure 6A). *L. brevis* CAAS 18052 and *L. fermentum* CAAS 18078 also did not significantly affect hepatic CYP7A1 levels in hamsters (*p* > 0.05) (Figure 6B). However, *L. plantarum* CAAS 18008 significantly increased the hepatic CYP7A1 level in hamsters by 5.4 times (*p* < 0.05).

## 3. Discussion

Cholesterol removed from media by lactobacilli could be bound to surface of lactobacilli [11], incorporated into cellular membrane [25], or transferred into the cytoplasm of lactobacilli [26]. However, the removed cholesterol was mainly incorporated into the phospholipid bilayers of the cellular membrane of lactobacilli [27]. The incorporation of cholesterol has been reported to strengthen the cell envelope by increasing ratio of C to P and thereby enhance cellular resistance to enzymatic hydrolysis and ultrasonic damage [12]. Therefore, the incorporation of cholesterol into cellular membrane may benefit survival of lactobacilli in the gastrointestinal tract. However, up to now, no research has investigated whether the ingestion of lactobacilli capable of removing cholesterol will have a significant influence on serum cholesterol levels.

Cholesterol removal ability of lactobacilli markedly depends on their own growth status. Lactobacilli are able to remove a significant amount of cholesterol from media only in the growing status, and the resting and dead cells of lactobacilli generally have a very low ability to remove cholesterol [28]. Cholesterol absorption mainly occurs in the duodenum and upper jejunum, where dietary and biliary cholesterol is available for uptake from the intestinal lumen [29]. However, a higher concentration of bile salts occurs in these regions of the small intestine [30], which significantly inhibits the growth of intestinal bacteria, including lactobacilli. For these reasons, *L. brevis* CAAS 18052 struggled to remove a significant amount of cholesterol in the duodenum and upper jejunum of hamsters, although it showed greater cholesterol removal ability *in vitro*. This was an important reason why *L. brevis* CAAS 18052 did show significant cholesterol-lowering activity in hamsters.

NPC1L1 protein is a predicted polytopic membrane protein that plays a key role in the absorption of intestinal cholesterol [31]. NPC1L1 protein transfers free cholesterol into cells through vesicular endocytosis and it is highly expressed in the duodenum and upper jejunum of the small intestine [32]. In theory, the down-regulation of the small intestinal NPC1L1 protein has a potential to decrease the amount of cholesterol that is absorbed from the small intestine, thereby affecting serum cholesterol levels. The ingestion of *L. acidophilus* ATCC 4356 at a dose of 1 × 10^9^ CFU per day has been reported to lead to a significant decrease in serum LDL-cholesterol and total cholesterol levels in rats through the down-regulation of the small intestinal NPC1L1 protein by the strain ingested [33]. In contrast to this study, *L. fermentum* CAAS 18078 at the same dose did show a significant cholesterol-lowering activity in hamsters, although it showed the greater NPC1L1 protein down-regulation activity *in vitro*. This conflicting result may be due to the different properties of the strains and animal models used.

The down-regulation of NPC1L1 protein of Caco-2 observed in this study should be attributed to extracellular metabolites (soluble effector molecules) secreted by *L. fermentum* CAAS 18078 during the incubation [34]. Under the *in vitro* static incubation conditions, these soluble effector molecules could fully interact with the Caco-2 cells. However, under *in vivo* dynamic conditions, these soluble effector molecules struggled to fully interact with the epithelial cells of the duodenum and upper jejunum of hamster due to the intestinal peristalsis and interference of food components and intestinal bacteria. In addition, due to the growth inhibition of *L. fermentum* CAAS 18078 in the duodenum and upper jejunum of hamster by a higher concentration of bile salts, the ability of the strain to secrete the soluble effector molecules had to decline. These were also important reasons why *L. fermentum* CAAS 18078 did not show significant hypocholesterolemic activity in hamsters.

The catalysis of BSH is responsible for bile salt deconjugation by strains of lactobacilli. The bile salt deconjugation has become a profound mechanism on hypocholesterolemic effects of probiotic lactobacilli [35,36]. Fermented milk of BSH-active *L. reuteri* NCIMB 30242 has shown a significant hypocholesterolemic effect in hypercholesterolemic adults [10]. Oral administration of the immobilized BSH derived from *L. buchneri* ATCC 4005 led to a significant decrease in serum total cholesterol level by 58% in rats fed a cholesterol-rich diet [37]. In addition, the fermented milk prepared by wild-type *L. casei* F0822 significantly decrease serum LDL-cholesterol and total cholesterol levels in hamsters, whereas the fermented milk prepared by BSH-deficient mutant of *L. casei* F0822 did not showed the hypocholesterolemic effects in hamsters [38].

*L. plantarum* CAAS 18008 showed the greater BSH activity in this study, which suggests that this strain was able to hydrolyze glycine- and/or taurine-conjugated bile salts in the intestinal tract of hamsters to release amino acids and free bile acids. The free bile acids are less soluble and poorly absorbed from the small intestine compared with their conjugated forms [39], which would increase fecal bile acid excretion levels [40]. To replace the lost bile acids, more new bile acids would be synthesized from cholesterol in the hepatic tissue of hamsters through catalysis of CYP7A1 and thereby would cause a significant decrease in the serum cholesterol levels in hamsters. In addition, the greater acid and bile tolerance ability also promoted survival of *L. plantarum* CAAS 18008 in the gastrointestinal tract and thereby enhanced its cholesterol-lowering activity.

## 4. Materials and Methods

### 4.1. Source and Maintenance of Lactic Acid Bacteria Strains

Twenty corn silage samples were collected from Shaanxi provine, China. The samples were serially diluted in sterile distilled water and the diluents (100 µL) were spread onto MRS agar plates (Oxoid, Basingstoke, Hampshire, UK). After anaerobic incubation for 72 h at 37 °C, typical colonies were selected from the plates and identified by both 16S rDNA sequencing and carbohydrate fermentation pattern using an API 50 CHL system (BioMeriéx, France) [41]. The isolates were genetically differentiated at the strain level by random amplification of polymorphic DNA-PCR using the primers M13, AB111, and AB106 [42]. A total of 85 strains of lactic acid bacteria were obtained and they consisted of 32 *L. plantarum* strains, 28 *L. brevis* strains, 20 *L. fermentum* strains, and 5 *E. faecium* strains. The cultures were stored in 30% glycerol at −86 °C. They were activated three times in MRS broth (Oxoid, Basingstoke, Hampshire, UK) prior to use.

### 4.2. Cholesterol Removal

Cholesterol removal ability of the lactic acid bacterial strains was measured according to the previous method [28] with minor modification. Briefly, water-soluble cholesterol (cholesteryl-polyethylene glycol 600 sebacate, Sigma-Aldrich, St. Louis, MO, USA) was added to the sterile MRS broth at a final concentration of approximately100 mg/L by filter sterilization (0.22 µm, Millipore, Bedford, MA, USA). The overnight cultures of lactic acid bacterial strains were inoculated into the broth with 1% (*v*/*v*) inoculum size and incubated anaerobically at 37 °C for 18 h. The cultures were centrifuged at 10,000 g for 15 min, and supernatants were taken for determining cholesterol concentrations by capillary gas chromatography [43].

### 4.3. NPC1L1 Proteiin Down-Regulation

NPC1L1 down-regulation by lactic acid bacterial strains was determined by the previous method [34] with several modifications. Briefly, the overnight cultures (16 h) of lactic acid bacterial strains were centrifuged at 10,000 g for 15 min, washed once with distilled water, and resuspended in Dulbecco’s Modified Eagle’s Medium (DMEM, Life Technologies, Carlsbad, CA, USA) at 1 × 10^8^ CFU/mL. The bacterial suspension (2 mL) was inoculated into monolayer Caco-2 cells, which were cultured on glass slide placed in six-well tissue culture plates, and incubated at 37 °C for 2 h. The medium was dumped out and the Caco-2 cells were washed twice with DMEM, and resuspended in phosphate buffer saline (PBS) after trypsinization to an absorbance of 1.0 at 600 nm. The cell suspension was mixed with the same volume of RIPA lysis buffer (Beyotime Institute of Biotechnology, Shanghai, China) and homogenized at 10,000 r/min for 10 min with an Ultra-Turrax T25 high-speed homogenizer (IKA Labortechnik, Staufen, Germany). The homogenates were used to determine NPC1L1 protein levels with a human NPC1L1 ELISA kit from AVIVA Systems Biology (San Diego, California, USA).

### 4.4. Bile Salt Deconjugation

The overnight cultures (16 h) of lactic acid bacterial strains were centrifuged at 10,000 g for 15 min, washed once with distilled water, and resuspended in MRS broth supplemented with a human bile salt mixture [44] (Sigma-Aldrich, total concentration of 4 mmol/L) at 1 × 10^8^ CFU/mL. The cultures were incubated anaerobically for 2 h at 37 °C, centrifuged at 10,000 g for 15 min, and the supernatants (1 mL) were drawn for determining the conjugated bile salt concentrations using ion-pair high-performance liquid chromatography [45].

### 4.5. Determination of Acid Tolerance

The overnight cultures (16 h) of lactic acid bacterial strains were centrifuged at 10,000 g for 15 min, washed once with distilled water, and resuspended in acidified MRS broth (pH 2.0, hydrochloric acid) at 1 × 10^8^ CFU/mL. The cultures were incubated for 2 h at 37 °C and one-milliliter sample was taken, and enumerated viable counts by plate pouring method. The acid tolerance ability of strains was calculated according to the following equation [46]:

(1) $$\mathrm{Acid}\;\mathrm{tolerance}\;\left(\%\right)=\frac{\log{\;N}_{1}}{\log{\;N}_{0}}\;\times \;100\;$$

where N_0_ is the total viable count before the incubation (CFU/mL) and N_1_ is the total viable count after the 2-h incubation (CFU/mL).

### 4.6. Determination of Bile Tolerance

Bile tolerance ability of the cultures of lactic acid bacterial strains was measured according to the previous method [45]. Briefly, the overnight cultures (16 h) of lactic acid bacterial strains were inoculated (0.01‰, *v*/*v*) into 1/2 strength buffered MRS broth (pH 7.3, 0.1 mol/L sodium phosphate) supplemented with and without 0.3% (*w*/*v*) oxgall (BD Difco, Sparks, MD, USA), and incubated for 12 h at 37 °C under anaerobic conditions. One-milliliter sample was taken, and enumerated viable counts by plate pouring method. The bile tolerance ability of the cultures was calculated based on the propagation rate in the presence of bile according to the following equation:

(2) $$\mathrm{Bile}\;\mathrm{tolerance}\;\left(\%\right)=\frac{\log_{2}\frac{N_{1}}{N_{0}}}{\log_{2}\frac{N_{2}}{N_{0}}}\times 100$$

where N_0_ is the viable counts before the incubation in the broth (CFU/mL) and N_1_ and N_2_ are the viable counts after the 12-h incubation in the broth with and without oxgall, respectively.

### 4.7. Determination of Adhesion Ability

The overnight cultures (16 h) of lactic acid bacterial strains were centrifuged at 10,000 g for 15 min, washed once with distilled water, and resuspended in DMEM at 1 × 10^8^ CFU/mL. The suspensions (2 mL) were inoculated into monolayer Caco-2 cells, which were cultured on glass slide placed in six-well tissue culture plates, and incubated at 37 °C for 1 h. The cells were washed three times with DMEM to remove unbound bacteria, fixed with 2 mL of methanol, stained with 2 mL of Giemsa stain solution (1:20) (Sigma-Aldrich) [47]. The adhesion ability of lactic acid bacterial strains was expressed as the adherent bacterial counts per 100 Caco-2 cells.

### 4.8. Animal Feeding Trial

Six-week-old male Syrian hamsters were obtained from Beijing Vital River Laboratory Animal Technology Company (China). Hamsters were individually housed in a room kept at a 22 ± 2 °C temperature, a 60 ± 5% humidity, and a 12-h light-dark cycle. All animal experiments were conducted under the Guide for Care and Use of Laboratory Animals [48] and the procedures involving animals were approved by Animal Ethical Committee of China Agricultural University (No. CAU20171020-3, 20 October, 2017).

Animals were randomly divided into 4 groups of 8 hamsters. All animals were fed a high-cholesterol diet (0.4 % cholesterol in AIN 93 M diet) during a 28-d feeding period [49]. First group (high-cholesterol control) was given 1-mL distilled water daily by gavage, and the other three groups were given 1-mL bacterial cell suspensions of *L. brevis* CAAS 18052, *L. fermentum* CAAS18087 and *L. plantarum* CAAS 18008 (1 × 109 CFU/mL each) daily by gavage, respectively. All animals were allowed free access to feed and water during the feeding period. 

### 4.9. Determination of Serum and Hepatic Cholesterol Levels

Hamsters were fasted for 12 h and whole blood was collected from the retro-orbital plexus, centrifuged at 3000 g for 15 min for separating serum. The obtained serum samples were used to determine low-density lipoprotein (LDL)-cholesterol, high-density lipoprotein (HDL)-cholesterol and total cholesterol levels by enzymatic colorimetry using a Synchron LX20 automated biochemical analyzer (Beckman Coulter, Fullerton, CA, USA) with commercial kits from Nanjing Jiancheng Bioengineering Institute (China). For hepatic cholesterol analysis, liver homogenates were extracted twice with a binary mixed solvent of chloroform and methanol (2:1, *v*/*v*) and the lower-layer organic phase (chloroform) was combined for determining total cholesterol levels by gas chromatography [42] using a 7890 A gas chromatograph equipped with a flame ionization detector and a HP-5 fused silica capillary column (30 m × 0.25 mm; film thickness 0.25 μm) (Agilent Technologies, Inc., Wilmington, Delaware, USA) set at a flow rate of carrier gas (N_2_) of 1 mL/min.

### 4.10. Determination of Fecal Neutral and Acidic Sterols

Feces were collected over the last 3 days and dehydrated by lyophilization. The dried fecal samples (100 mg) were extracted twice with 2-mL absolute ethyl alcohol at 50 °C. The combined extracts were used to determine enzymatically total bile acid levels using a Synchron LX20 automated biochemical analyzer with a commercial kit from Nanjing Jiancheng Bioengineering Institute (China) and determine fecal neutral sterols (cholesterol, coprostanol, and cholestane) by gas chromatography-mass spectrometry [18] using a 7890 A gas chromatograph fitted with a 5975-C mass spectrometry detector (electron impact ion source) and a HP-5 MS fused silica capillary column (30 m × 0.25 mm; film thickness 0.25 μm) (Agilent Technologies, Inc., Wilmington, Delaware, USA) set at a flow rate of carrier gas (H_e_) of 1 mL/min.

### 4.11. Determination of Small Intestinal NPC1L1 Protein and Hepatic Cholesterol-7α-Hydroxylase (CYP7A1)

The whole small intestine and liver were homogenized in ice-cold RIPA lysis buffer with an Ultra-Turrax T25 homogenizer for 10 min at 9000 r/min. The homogenates were centrifuged at 8000 g for 15 min and the supernatants were drawn for analyzing small intestinal NPC1L1 protein and hepatic CYP7A1 levels with commercial hamster NPC1L1 and CYP7A1 ELISA kits from Haling Biological Technology Company (Shanghai, China), respectively [38].

### 4.12. Statistical Analysis

All experiments were repeated three times except the animal feeding trial (*n* = 8) and data were expressed as the mean ± standard deviation. Statistical analysis was performed using SPSS software (version 24.0, IBM Corporation, Armonk, NY, USA). Statistical differences between the means were analyzed by one-way analysis of variance followed by Duncan’s multiple-range test. A difference of *p* < 0.05 was considered statistically significant.

## 5. Conclusions

The strains showing the greater cholesterol removal and NPC1L1 protein down-regulation activity *in vitro* had no significant effects on serum cholesterol levels in hamsters, whereas *L. plantarum* CAAS 18008 showing the greater bile salt deconjugation ability *in vitro* significantly decreased serum HDL-cholesterol, total cholesterol, and hepatic total cholesterol levels in hamsters. 

## Figures and Tables

**Table 1:** Acid tolerance, bile tolerance, and adhesion ability of the selected strains.

Hypothesized Pathway	Strain	Acid Tolerance (%)	Bile Tolerance (%)	Adhesion (Bacterial Counts /100 cells)
Greater cholesterol removal	*L. plantarum* CAAS 18010	61.6 ± 2.2 ^b^	NG	282 ± 30 ^a^
	*L. plantarum* CAAS 18021	33.6 ± 4.1 ^f^	83.6 ± 5.5 ^a^	281 ± 19 ^a^
	*L. brevis* CAAS 18035	66.3 ± 2.0 ^ab^	41.5 ± 3.8 ^h^	251 ± 10 ^ab^
	*L. brevis* CAAS 18041	65.9 ± 3.7 ^ab^	81.9 ± 5.6 ^ab^	228± 34 ^bc^
	*L. brevis* CAAS 18052	65.2 ± 3.9 ^ab^	75.8 ± 2.8 ^bc^	224 ± 23 ^bc^
	*L. fermentum* CAAS 18066	22.5 ± 1.0 ^h^	54.6 ± 4.1 ^g^	213± 28 ^bc^
	*L. fermentum* CAAS 18069	55.5 ± 3.2 ^c^	NG	107 ± 19 ^e^
	*L. fermentum* CAAS 18074	21.7 ± 3.0 ^h^	76.0 ± 5.1 ^bc^	241 ± 26 ^ab^
NPC1L1 protein down-regulation	*L. fermentum* CAAS 18062	30.8 ± 2.4 ^fg^	75.5 ± 4.2 ^bc^	245 ± 27 ^ab^
	*L. fermentum* CAAS 18067	49.9 ± 3.8 ^cd^	54.6 ± 3.6 ^g^	106 ± 19 ^e^
	*L. fermentum* CAAS 18070	63.4± 2.9 ^ab^	NG	151 ± 23 ^d^
	*L. fermentum* CAAS 18078	61.2 ± 3.4 ^b^	65.8 ± 2.3 ^de^	240 ± 22 ^abc^
Greater bile salt deconjugation	*L. plantarum* CAAS 18004	61.5 ± 3.4 ^b^	NG	205 ± 26 ^bc^
	*L. plantarum* CAAS 18008	68.5 ± 3.0 ^a^	75.7 ± 4.2 ^bc^	241± 14 ^ab^
	*L. plantarum* CAAS 18017	55.5 ± 4.3 ^c^	80.7 ± 4.7 ^ab^	93 ± 20 ^e^
	*L. plantarum* CAAS 18025	40.2 ± 3.0 ^e^	63.5 ± 4.2 ^ef^	101 ± 24 ^e^
	*L. brevis* CAAS 18038	27.0 ± 2.6 ^gh^	71.8 ± 2.9 ^cd^	207± 29 ^bc^
	*L. brevis* CAAS 18044	63.9 ± 3.9 ^ab^	74.8 ± 4.3 ^bc^	194 ± 34 ^c^
	*E. faecium* CAAS 18083	45.5 ± 3.3 ^de^	57.5 ± 1.7 ^fg^	121 ± 26 ^de^

^a–g^ Means in the same column not sharing a common superscript letter differed significantly (*p* < 0.05). A NG sign indicates that the strains did not grow in the presence of 0.3% oxgall.
